# Elevated fetal steroidogenic activity in autism

**DOI:** 10.1038/mp.2014.48

**Published:** 2014-06-03

**Authors:** S Baron-Cohen, B Auyeung, B Nørgaard-Pedersen, D M Hougaard, M W Abdallah, L Melgaard, A S Cohen, B Chakrabarti, L Ruta, M V Lombardo

**Affiliations:** 1 Autism Research Centre, Department of Psychiatry, University of Cambridge, Cambridge, UK; 2 Department of Psychology, University of Edinburgh, Edinburgh, UK; 3 Department of Clinical Biochemistry and Immunology, Statens Serum Institute Copenhagen, Copenhagen, Denmark; 4 Department of Child and Adolescent Neuropsychiatry, Rostock University Medical Centre, Rostock, Germany; 5 Section for Epidemiology, Department of Public Health, Aarhus University, Aarhus, Denmark; 6 Centre for Integrative Neuroscience and Neurodynamics, School of Psychology and Clinical Language Sciences, University of Reading, Reading, UK; 7 Division of Child Neurology and Psychiatry, Stella Maris Scientific Institute, Pisa, Italy; 8 Department of Psychology, University of Cyprus, Nicosia, Cyprus; 9 Centre for Applied Neuroscience, University of Cyprus, Nicosia, Cyprus

## Abstract

Autism affects males more than females, giving rise to the idea that the influence of steroid hormones on early fetal brain development may be one important early biological risk factor. Utilizing the Danish Historic Birth Cohort and Danish Psychiatric Central Register, we identified all amniotic fluid samples of males born between 1993 and 1999 who later received ICD-10 (International Classification of Diseases, 10th Revision) diagnoses of autism, Asperger syndrome or PDD-NOS (pervasive developmental disorder not otherwise specified) (*n*=128) compared with matched typically developing controls. Concentration levels of Δ4 sex steroids (progesterone, 17α-hydroxy-progesterone, androstenedione and testosterone) and cortisol were measured with liquid chromatography tandem mass spectrometry. All hormones were positively associated with each other and principal component analysis confirmed that one generalized latent steroidogenic factor was driving much of the variation in the data. The autism group showed elevations across all hormones on this latent generalized steroidogenic factor (Cohen’s *d*=0.37, *P*=0.0009) and this elevation was uniform across ICD-10 diagnostic label. These results provide the first direct evidence of elevated fetal steroidogenic activity in autism. Such elevations may be important as epigenetic fetal programming mechanisms and may interact with other important pathophysiological factors in autism.

## Introduction

Autism is characterized by early developmental difficulties in social interaction and communication, alongside highly repetitive stereotyped behavior and/or restricted interests. It affects approximately 1% of the population, and males are diagnosed more often than females.^1^ While changing diagnostic practice over time and other social variables likely partly contribute to this male bias, biological factors related to the sex chromosomes and/or fetal sex steroids may also have important roles.^2,3^ Given their capacity to exert epigenetic fetal programming influence during early critical periods of brain development,^4, 5, 6, 7^ steroid hormones are well-positioned as an important early fetal environmental factor that may interact with other risk factors for autism and other atypical neurodevelopmental conditions that asymmetrically affect the sexes.^2,8, 9, 10, 11^

Prior work has shown that one prominent sex steroid, testosterone, has wide-ranging influence on phenotypic variability related to autism. For example, fetal testosterone influences individual differences in typical development in eye contact, vocabulary size, restricted interests, mentalizing, empathy, systemizing, attention to detail and autistic traits.^11^ Emerging neuroimaging evidence also supports a role for fetal testosterone affecting individual differences in structural and functional brain development in directions congruent with patterns of sexual dimorphism, and in brain regions known to develop atypically in autism and other sex-biased developmental psychopathologic conditions.^2,9,12^ The ‘extreme male brain’ theory of autism,^3^ which is primarily an account about the cognitive phenotype of autism, makes mechanistic predictions about the role of elevated fetal sex steroids such as testosterone in the development of autism. However, the extreme male brain theory does not exclude the role of other mechanisms such as the role of sex chromosome effects or the role of other steroid hormones proximal to testosterone in biosynthesis pathways (e.g., estradiol, progesterone and cortisol). Therefore, although prior work in relation to autism has mainly tested fetal testosterone, it may be that the actions of testosterone and other precursor steroid hormones within the same biosynthesis pathway (e.g., the Δ4 sex steroid pathway) all have significant roles during fetal development in autism. For example, prior genetic association evidence has found a role for cytochrome *P*450-containing enzymes in autism (e.g., CYP17A1, CYP19A1 and CYP11B1).^13^ These enzymes are critical for the conversion of different hormones along the Δ4 pathway that lead up to testosterone (e.g., progesterone, 17α-hydroxy-progesterone and androstenedione) as well as for conversion of Δ4 steroids such as progesterone and 17α-hydroxy-progesterone toward cortisol (see Supplementary Figure 1). Thus, in this study we test the role of not only fetal testosterone but also precursor steroids within the Δ4 pathway, as well as the non-Δ4 steroid hormone cortisol, as potential mechanisms that are elevated during fetal development in autism.

However, given the 1% prevalence rate, previous studies have been too small to obtain a sample size large enough to test the hypothesis of whether fetal steroid activity is elevated in those who later receive a clinical diagnosis of autism. In the present study, we overcome this limitation by examining a large biobank of amniocentesis samples from the Danish Historic Birth Cohort (HBC), archived at the Statens Serum Institute in Copenhagen, Denmark. The HBC has stored amniocentesis, bloodspots and maternal serum samples from the Danish population since the late 1970s and includes more than 100 000 samples. Using samples of individuals born between 1993 and 1999, we linked samples in the HBC to the Danish Psychiatric Central Register to identify all amniotic fluid samples of individuals who went on to later receive an ICD-10 (International Classification of Diseases, 10th Revision) diagnosis of childhood autism, Asperger syndrome or PDD-NOS (pervasive developmental disorder not otherwise specified). We measured the concentration levels of sex steroids along the CYP17-mediated Δ4 sex steroid biosynthesis pathway (i.e., progesterone, 17α-hydroxy-progesterone, androstenedione and testosterone). We also measured the glucocorticoid steroid hormone cortisol, because it is well known as a fetal programming mechanism associated with a number of atypical phenotypes such as anxiety disorder and attention deficit-hyperactivity disorder^10^ that commonly co-occur with autism.^14^ Cortisol is also positively correlated to sex steroid levels such as testosterone in both amniotic fluid and fetal plasma,^15,16^ but its position outside the Δ4 sex steroid pathway (see Supplementary Figure 1) allows for testing neuroendocrine specificity of any potentially atypical fetal steroidogenic activity in autism. Given prior work,^2,3^ we predicted there would be elevated levels of steroidogenic activity across all subgroups on the autism spectrum and across all Δ4 sex steroid hormones, but predicted no difference in cortisol levels.

## Materials and Methods

### Participants

**Figure 1:**
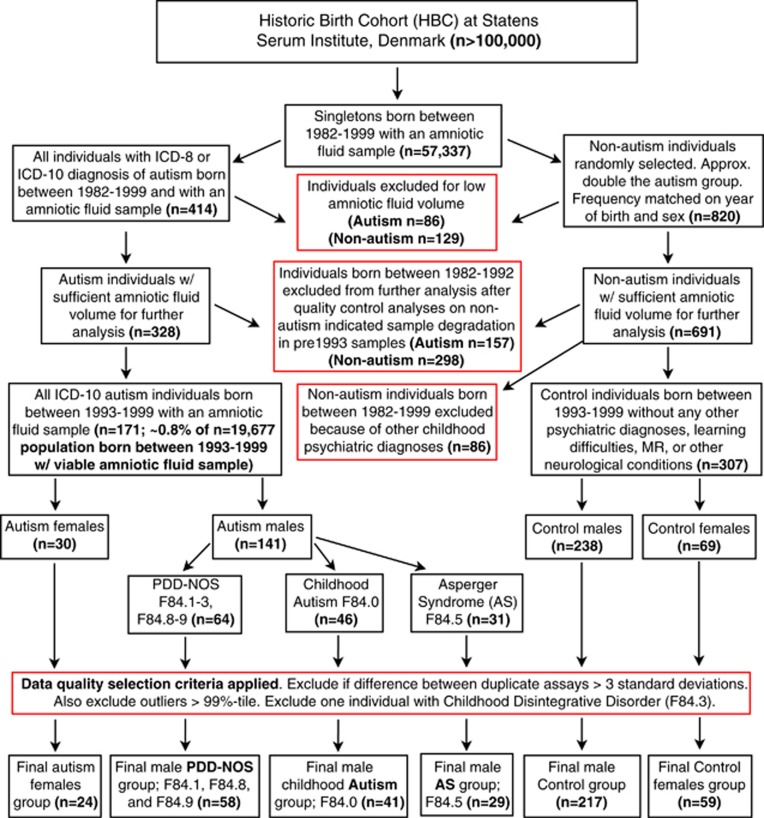
Flowchart depicting population, selection criteria and stratification into samples used for final analyses. This flowchart shows the original population sizes within the Historic Birth Cohort (HBC) and the population from which our study was drawn from (individuals born between 1993 and 1999). Red boxes indicate steps where data were selected or excluded. AS, Asperger syndrome; PDD-NOS, pervasive developmental disorder not otherwise specified; MR, mental retardation.

Participants were selected from a population of singleton births between the years 1993 and 1999 (*n*=19 677) whose amniotic fluid samples were stored within the HBC.^17^ Our study is restricted to individuals born between 1993 and 1999 because quality control analyses on typically developing control individuals born between 1982 and 1992 showed marked signs of changes in concentration levels compared with individuals born between 1993 and 1999 that are likely because of evaporation and a systematic change in the caps used for sealing the tubes that begun in 1993 (see Supplementary Information). Between November 2009 to January 2010, we searched the HBC and Danish Psychiatric Central Register and identified all cases with an ICD-10 diagnosis of PDD codes (F84.0: childhood autism; F84.5: Asperger syndrome; F84.1: atypical autism; F84.8: other PDDs; F84.9: PDD, unspecified). After data-quality filtering was applied (see Figure 1), the number of total female autism cases was small (*n*=24) and preliminary analysis of non-hormonal variables indicated that female autism cases had significantly older fathers at birth (autism mean=37.45 years, s.d.=7.20; control mean=34.50 years, s.d.=5.52; *t*(81)=2.01, *P*=0.04) and weighed less than controls (autism mean=3142.8 g, s.d.=490.73; control mean=3404.4 g, s.d.=557.41; *t*(81)=2.00, *P*=0.04). Such problems were not apparent for male autism cases and thus all analyses were conducted on males only. Typically developing controls came from randomly selected sample of individuals from the HBC with no known neuropsychiatric diagnoses. The final sample sizes in all analyses are *n*=217 controls and *n*=128 autism. Among the autism group, individuals comprised the following ICD-10 subdiagnoses: *n*=41, F84.0: childhood autism; *n*=29, F84.5: Asperger syndrome; *n*=4, F84.1: atypical autism; *n*=15, F84.8: other PDDs; *n*=39, F84.9: PDD, unspecified. However, all analyses treated autism as one group rather than splitting among subdiagnostic labels.

### Sample storage and hormone assays

Amniotic fluid samples in the HBC were centrifuged immediately after collection and the supernatants were kept at −20 °C, following the routine procedure for storing and handling biologic materials at the Statens Serum Institute.^18^ Hormone assays took place at the beginning of 2010 and consisted of liquid chromatography tandem mass spectrometry of concentration levels of progesterone, 17α-hydroxy-progesterone, androstenedione, testosterone and cortisol. We computed a variable for ‘storage time’ based on the difference between time at which the assays were carried out and an individual’s year of birth. All calibrators, controls, internal standards and μ-titer plates were purchased from Perkin-Elmer (Waltham, MA, USA) via their CHS Steroid Profiling Kit for mass spectrometry (Perkin-Elmer). Formic acid was purchased from Merck (Darmstadt, Germany). Ammonium acetate and zinc sulfate heptahydrate were purchased from Sigma-Aldrich (St Louis, MO, USA). Water was purified using a Millipore water purification unit (Millipore, Billerica, MA, USA).

The liquid chromatography tandem mass spectrometry system consisted of an Aria TLX2 system (Thermo Fisher Scientific, Waltham MA, USA) with two Agilent 1100 binary pumps and two Agilent 1200 quaternary pumps (Agilent, Santa Clara, CA, USA) connected to a Thermo TSQ Ultra triple quarupole mass spectrometer equipped with a atmospheric pressure chemical ionization ion source. Extraction was performed using a Cyclone P 0.5 × 50 mm^2^ TurboFlow column (Thermo) and analytical separation was achieved using Kinetix 2.6 μ 2.1 × 50 mm^2^ C18 columns (Phenomenex, Torrance, CA, USA). The following solvents were used: loading buffer A was composed of 5% methanol, 95% water containing and 0.1% formic acid (LA), loading buffer B was composed 100% methanol containing 10 mmol l^−1^ ammonium acetate (LB), eluting buffer A was comosed of 2% methanol and 98% water (EA) and eluting buffer B (EB) was identical to LB. The analytes were trapped on the TurboFlow extraction column using 100 LA at a flow rate of 1.5 ml min^−1^. After 60 s, the analytes were transferred to the analytical column using 100 μl of LB at a flow rate of 0.15 ml min^−1^ for a duration of 60 s. The transferred analytes were concurrently diluted using 60% EA and 40% EB at a flow rate of 0.3 ml min^−1^. Subsequently, the analytes were separated before mass spectrometry analysis using gradient elution from 53% EA/47% EB to 35.5 EA/64.5% EB at a flow rate of 0.4 ml min^−1^.

The mass spectrometry system was operated in a positive mode using atmospheric pressure chemical ionization. The following source settings were used: discharge current 5 mA, vaporizer temperature 450 °C, sheath gas pressure 25 a.u., ion sweep gas pressure 0 a.u., auxiliary gas pressure 25 a.u., capillary temperature 300 °C and collision pressure 1.5 mTorr. Collision voltages and T-lens voltages were optimized for each transition. For the analytes, the following transitions were used: progesterone (315.1/97,315.1/109), 17α-hydroxy-progesterone (331.1/97, 331.1/109), androstenedione (287.1/97, 287.1/109), testosterone (289.1/97, 289.1/109) and cortisol (363.1/97, 363.2/121). For the internal standards, the following transitions were used: progesterone-d_9_ (324.2/100, 324.2/113), 17α-hydroxy-progesterone-d_8_ (339.2/100, 339.2/113), androstenedione-d_5_ (292.2/100, 292.2/113) and testosterone-d_5_ (294.2.1/100, 294.2/113) and cortisol-d_3_ (366.3/97, 366.3/121).

Calibrators and controls were reconstituted with water according to the kit insert. A deproteinizing solution consisting of 18 g l^−1^ zinc sulfate heptahydrate in 80% methanol/20% water containing internal standard was prepared. Fifty microliters of amniotic fluid samples, calibrators and controls were pipetted onto a 98-well μ-titer plate. One hundred microliters of  deproteinizing solution was added to precipitate proteins from the samples. The plate was sealed with a silicone plate map and was incubated at −20 °C for 4 h. The plate was centrifuged at 4000 r.p.m. and the supernatant was transferred to a new μ-titer plate. The plate was covered with aluminum sealing foil and was placed in the autosampler prior analysis.

### Statistical analysis

Before data analysis, we first implemented data-quality selection criteria. We excluded all individuals where duplicate assays resulted in a difference >3 s.d. from the entire sample. We also excluded outliers whose concentration levels were greater than the 99th percentile defined based on the entire sample born between 1993 and 1999. These outliers were determined to be primarily artifactual in origin, given that testosterone concentrations for these outliers were far above the maximum concentration values ever observed in prior studies of fetal testosterone either in our or independent laboratories.^11,16^ Given that changes in sample concentration due to storage-related factors are well-known issues^19^ (see Supplementary Information), the exclusion of such extreme outliers helped guard against this artifactual influence.

To investigate associations between hormones in each group, we computed the correlation matrices estimated via robust regression to allow for insensitivity to bivariate outlying data points. We also ran principal component analysis (PCA) implemented with pca.m in Matlab R2012B after preprocessing the dataset via *z*-scoring. PCA was conducted to inspect the latent structure of the dataset and to confirm that one latent variable related to generalized steroidogenic activity was driving a large proportion of the variance in the dataset. After running PCA, we ran our main hypothesis tests as one-way analysis of variances (ANOVAs) on the PCA scores (5 ANOVAs; one per component) and evaluated the results at a Bonferroni-corrected α-level of *P*<0.01. To check that maternal age, paternal age, birth weight, gestational age at amniocentesis and storage time did not contribute to confounding influences on the hypothesis tests, we re-ran hypothesis tests as ANCOVAs with these variables included as covariates.

At a descriptive level of analysis, we next assessed the patterning of the group differences along the first principal component across all hormones and all ICD-10 autism subdiagnoses. To achieve this, we reconstructed the data in its original space by multiplying the first principal component scores with its loading coefficients (i.e., matrix multiplication of the first PC scores against the transposed vector of the first PC loading coefficients) and then reversed the *z*-scoring process to have the data back on its original scale. Once this was done, we used dot plots to plot individual data points and also plotted the mean and 95% confidence intervals on top of the individual dots for all hormones separately and all ICD-10 subgroups separately. No further statistical tests were carried out at this point and this process is merely for descriptive purposes, to illustrate how each hormone and ICD-10 subgroup contribute to the differences within the first principal component.

While we view the hypothesis tests on the PCA scores as a more parsimonious approach to the dataset, we also ran hypothesis tests on the raw data to verify whether similar inferences would remain without having PCA transform the data along latent variables. Here, we implemented a multivariate analysis of variance on raw hormone concentration levels and re-ran the analysis again after including maternal age, paternal age, birth weight, gestational age at amniocentesis and storage time as covariates.

## Results

Ninety-five percent or more of individuals from each group had Apgar scores >6 at 5 min after birth, indicating that almost all participants in this study were classified as healthy at birth. Non-hormonal variables of interest such as maternal and paternal age, birth weight and gestational age at amniocentesis did not differ between groups. Furthermore, ruling out storage time as a possible confound, we found that groups did not differ in terms how long samples were kept in storage (see Table 1). Demonstrating that these factors do not differ on average between groups is important given that many have prior links to autism, as well as their general importance as health-related fetal programming influences. However, although there were no significant between-group differences on any of these variables, we continued to view these variables as covariates in further analyses.

**Figure 2:**
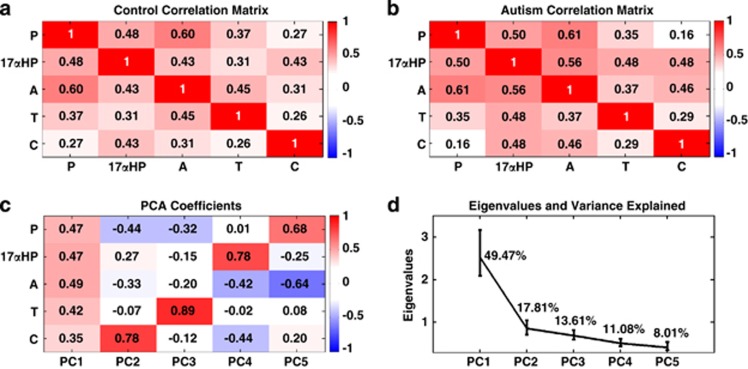
Associations between steroid hormones and latent steroidogenic factors. (**a** and** b**) Correlation matrices depicting the relationships between all hormones measured in the study for the typically developing the control group (**a**) and the autism group (**b**). (**c**) PCA loading coefficients for each hormone on each component. (**d**) A scree plot of the eigenvalues for each component and the percentage of variation each component explains. Error bars in (**d**) represent the 95% bias-corrected and accelerated bootstrap confidence intervals for the eigenvalues estimated from 10 000 bootstrap resamples*.*  A, androstenedione; C, cortisol; 17αHP, 17α-hydroxy-progesterone;  P, progesterone; PCA, principal component analysis; T, testosterone.

Given the close proximity between the measured hormones within steroidogenic biosynthesis pathways and prior work suggesting positive relationships between steroid hormones, such as testosterone and cortisol in fetal development,^15,16^ we examined relationships between all measured hormones. Irrespective of group membership, concentration levels of all hormones were positively related (Figures 2a and b). This suggests one underlying latent ‘steroidogenic’ factor driving concentration levels in the amniotic fluid. Quantitative confirmation of this hypothesis can be seen in the results of a PCA, which revealed one prominent component explaining 49.47% of the variance in the data, and all five hormones loaded relatively equally onto it. The other four components explained substantially smaller amounts of variance (<17%) and loaded predominantly onto two or three hormones (Figures 2c and d). A scree plot of the eigenvalues (Figure 2d) shows an ‘elbow’ at component 2 and eigenvalues are all below 1 for components 2–5. Given that PCA allowed for parsimonious unsupervised separation of various sources of variation in the dataset, we went ahead with main tests of our hypotheses based on the PCA scores for each component.

**Figure 3:**
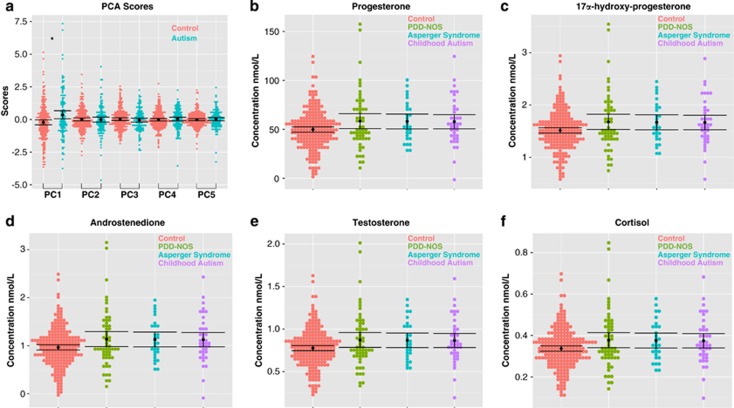
Group differences in amniotic fluid hormone concentration levels across each group. PCA scores for each component are shown in  (**a**). The mean is shown as a black dot, and the error bars represent the 95% confidence intervals. Underneath the mean and error bars are dots for each individual in the sample (controls, pink; autism, light blue). *A group difference (passing the Bonferroni-corrected *α* of *P*<0.01) in the first principal component (PC) (Cohen’s *d*=0.37, *P*=0.0009), where scores are elevated in autism compared with controls. (**b**–**f**) The hormone concentrations after the data are reconstructed from the first PC scores and loadings. These bar graphs enable viewing of the patterns across each hormone and across each ICD-10 autism subgroup, to describe what contributes to the group differences in the latent steroidogenic factor represented by the first PC. In these plots, the mean is shown as a black dot and the error bars represent the 95% confidence intervals. Underneath the mean and error bars are dots for each individual in the sample (controls, pink; PDD-NOS, green; Asperger syndrome (AS), light blue; childhood autism, purple). PCA, principal component analysis;  PDD-NOS, pervasive developmental disorder not otherwise specified.

One-way ANOVAs on the PCA scores for each component (coding all individuals with autism the same irrespective of their ICD-10 subgroup) revealed that only the first principal component, reflecting a latent generalized steroidogenic factor, was significantly different between groups (F(1,343)=11.18, *P*=0.000915, Cohen’s *d*=0.37), and this was driven by elevations in the autism group (Figure 3a). No other components revealed any between-group differences (PC2: F(1,343)=0.025, *P*=0.87, Cohen’s *d*=−0.01; PC3: F(1,343)=0.73, *P*=0.39, Cohen’s *d*=−0.09; PC4: F(1,343)=0.42, *P*=0.51, Cohen’s *d*=0.07; PC5: F(1,343)=0.30, *P*=0.58, Cohen’s *d*=0.06). Including other non-hormonal variables such as paternal age, maternal age, birth weight, gestational age at the time of amniocentesis and storage time as covariates did not change any of the inferences (PC1: F(1,334)=11.01, *P*=0.001; PC2: F(1,334)=0.27, *P*=0.59; PC3: F(1,334)=0.79, *P*=0.37; PC4: F(1,334)=0.70, *P*=0.40; PC5: F(1,334)=0.18, *P*=0.67). Furthermore, similar inferences are obtained using a multivariate analysis of variance on raw concentration levels (Wilk’s *Λ*=0.964, *P*=0.029) and when including non-hormonal covariates (Wilk’s *Λ*=0.962, *P*=0.023) (see Table 2 for descriptive statistics on the raw concentration levels).

We next went further into describing the nature of differences in the ‘steroidogenic’ component by reconstructing the data in its original space from the coefficients and scores from the first principal component. This reconstruction allowed us to  visualize descriptively the patterns of data from this steroidogenic component for each hormone separately, as well as for each ICD-10-defined autism subgroup. Elevations are prominent across all hormones, and similar irrespective of ICD-10 autism subgroup (Figures 3b–f).

## Discussion

This is the first study to test directly the prediction that fetal steroidogenic activity is elevated in autism. We find that amniotic fluid steroid hormones are elevated in those who later received diagnoses on the autism spectrum. Rather than the abnormality being restricted to a specific steroid hormone, a latent steroidogenic factor is elevated, which includes all hormones in the Δ4 pathway, as well as cortisol. This observation suggests dysregulation of pathways mediated by cytochrome *P*450-containing enzymes that catalyze the conversion of hormones along the Δ4 and glucocorticoid pathways. Prior evidence pointing toward the importance of such enzymes was previously found via genetic associations between autism and single-nucleotide polymorphisms in *CYP17A1*, *CYP19A1* and *CYP11B1* genes.^13^

The source of elevated steroidogenic activity in the fetal development of autism was not tested in the current study, and more research will be needed to understand how different sources such as the fetus, mother, placenta or other environmental factors might contribute to such elevations. While there are robust sex differences in amniotic fluid testosterone (see meta-analysis in Supplementary Table 2 as well as documented sex differences in typically developing controls in this cohort in Supplementary Figure 7) that suggest that the fetus is a primary source,^20^ some research has documented correlations between amniotic fluid levels and maternal plasma that would suggest that maternal–placental–fetal transfer is another possible source.^16,21,22^ Recent work also suggests that the placenta is capable of synthesizing sex steroids *de novo* from maternal substrates (cholesterol), maternal or fetal prohormones and steroid precursors.^23^ Active steroids produced in the placental unit can be released into both the maternal and fetal compartments to maintain the pregnancy and fetal development.^24^ In addition, endocrine disrupting compounds may be another source of environmentally mediated influence on the early fetal hormonal environment and autism.^25^ Each of these sources require further investigation to determine how such influences might affect fetal development in autism.

Fetal steroidogenic abnormalities in autism are also important because of their known interactions with other findings in the early development of autism. Steroids and their receptors act as epigenetic fetal programming influences on early brain development. Through their nuclear hormone receptors, steroids can alter gene expression via direct or indirect influence on multiple epigenetic processes such as histone acetylation, DNA methylation and have transcriptional and post-transcriptional effects on noncoding mRNAs such as microRNAs. Furthermore, during early sensitive periods of brain development, there are sex differences in DNA methylation, methyl-binding proteins, chromatin modifications and microRNA expression, and these effects are mediated in part by early steroid hormone effects.^4, 5, 6^

Early steroid-mediated epigenetic influence can also be observed via genomic or non-genomic influences on neurodevelopmental processes/mechanisms thought to be atypical in autism, such as neurogenesis, apoptosis, neurotransmission and synapse formation/function and the immune system,^26,27^ and may also be important for understanding early pathophysiological mechanisms for autism.^2^ For example, steroid receptors upregulate Wnt signaling through their action on β-catenin,^28^ a molecule that has a crucial role in cell-to-cell adhesion. Atypical cell-to-cell adhesion could potentially underlie minicolumnopathy^29^ and other abnormalities at the synapse in autism.^30^ Interestingly, a prominent autism-candidate gene involved in synapse formation, *NLGN4X*, shows sex-biased differential exon usage that is particularly pronounced during fetal development.^31^ Sex steroids also influence early developmental changes in GABAergic signaling.^32^ Given known abnormalities in GABAergic signaling in autism, elevations in early fetal sex steroids may modulate GABAergic control of the excitatory/inhibitory balance in the developing autistic brain.^33,34^ Steroid hormones also interact with the immune system and a striking example of this is how early sex steroids influence sexual dimorphism in the brain and behavior through their effects on microglia activation.^26^ Many studies have observed a wide array of atypicalities in the immune system in autism.^35^ Converging with the current results, we previously found atypical chemokine and cytokine profiles in both amniotic fluid and neonatal bloodspots of individuals with a diagnosis of autism in the HBC.^36, 37, 38^ These arguments point to the need for further work investigating interactions between fetal programming effects of steroid hormones and other early pathophysiological mechanisms in autism.

The current results may also be relevant to the literature on prenatal stress and autism. We found that cortisol, a biomarker typically associated with stress, is elevated early in the fetal development of autism. Studies of prenatal stress in autism have been mixed and vary substantially in methodological detail.^39,40^ While the current results may suggest a link between prenatal stress and autism via heightened fetal cortisol, it is unclear if the association here is due to heightened prenatal stress or is driven by a more primary fetal sex steroid influence that has a side effect of boosting fetal cortisol levels. Furthermore, although cortisol is a prominent stress hormone at later points in development, it may have different functions at earlier stages of brain development. As mentioned earlier, unlike in adulthood, cortisol and sex steroids such as testosterone are positively related to each other in fetal development^15,16^ and this may suggest a more varied role for cortisol in early brain development. The current work is consistent with prior suggestions of interactions between the fetal hypothalamic-pituitary-adrenal and hypothalamic-pituitary-gonadal axes, and their known fetal programming effects on later atypical neurodevelopmental phenotypes.^41^

From a clinical standpoint, the current results say nothing about the potential for such data as a prospective prenatal test of autism risk. The current findings are based on average-level group differences that are useful for hypothesis testing but are not directly applicable for prediction of individual diagnoses. Further work examining the predictive utility of these and other fetal markers is necessary to evaluate their implications for clinical screening. Finally, the present results should not be used as empirical justification for treatments that target sex steroids. The androgen-blocker Lupron has already been inappropriately offered^42^ as a treatment for autism. Aside from the ethical and safety issues this type of treatment raises, there is little conceptual rationale to justify its use in autism since an elevation in steroidogenic activity in *early* (fetal) development has no direct implications for the use of androgen-blocking drugs *later* in life. ‘Organizational’ effects of sex steroids on early fetal brain development tend to create permanent, irreversible changes in how the brain is organized at the cellular level^7,27^ and such treatments much later in life may be incapable of reversing such effects.

This study has certain limitations. First, data from central registries do not allow for collection of detailed information about clinical symptoms to validate diagnoses against gold-standard instruments. However, other studies have shown that within the Danish Psychiatric Central Register the validity ratio for childhood autism cases is 94% for 1990–1999.^43^ Second, hormones were assayed from a historic collection of amniocentesis samples stored over several years at −20 °C. Analyte concentration levels are known to change over time, especially when the time period stretches over 10 years.^19^ However, for the cohort analyzed in this study (born between 1993 and 1999), storage-time-dependent changes are unlikely to be a major issue. For instance, groups did not systematically differ in storage time and inclusion of this and other key variables as covariates did not change any inferences. Furthermore, concentration levels of testosterone were in line with levels typically observed across the literature and robust sex differences in testosterone were apparent as was the known prenatal surge peaking around gestational weeks 14–16 (see Supplementary Figure 7). In addition, the use of PCA-enabled unsupervised separation of various sources of variation in the data and highlighted the latent steroidogenic component. Even if variation due to storage-time-dependent changes affected the data from the first principal component of our analyses, there is no reason to suspect a bias toward inflating the concentration levels in one group relative to the other. Aside from PCA, a multivariate analysis of variance on the raw concentration levels also revealed elevations in the autism group. This suggests that while some storage-time-dependent changes are unavoidable in historic collections, it did not prevent our ability to test the main hypothesis. Finally, the prevalence estimate of autism within this amniocentesis cohort (0.8%) is consistent with prevalence estimates of autism in the Danish population during the same time period.^44^ Thus, although these results are derived from a selected sample, they may be representative of what would be expected in the general population. Nevertheless, future work on a sample more representative of the general population is necessary, as are tests of autism against other neurodevelopmental conditions that are asymmetrically affected across the sexes.

The current results provide initial support for a fetal steroid theory in explaining some aspects of early epigenetic risk for autism and/or other sex-biased neurodevelopmental conditions occurring during periods of fetal brain development. There are several promising directions for further testing this theory. Other steroid pathways (e.g., the Δ5 pathway, other glucocorticoids hormones), and particularly, estradiol, must be investigated in future work. Estradiol has long been known in non-human species to have potent masculinizing effects on the brain and behavioral development.^45^ In a prior study, we found genetic association evidence for autism in the *CYP19A1* gene, which codes for the protein aromatase that converts testosterone to estradiol. Gene expression levels of an autism-associated gene, retinoic acid-related orphan receptor-α (*RORA*), are lowered by testosterone and elevated by estradiol, and transcriptionally regulates several autism-associated genes including CYP19A1, thus affected aromatase levels.^46,47^ Although we were unable to test estradiol in the current study owing to lack of an automated liquid chromatography tandem mass spectrometry assay, we intend to investigate this in future work.

It is also necessary for future work to test if such elevations are specific to autism or are shared by other neurodevelopmental conditions with skewed sex ratios, and to test whether similar elevations exist in females with autism. Given the comorbidity of autism with other sex-biased conditions such as attention deficit-hyperactivity disorder, anxiety disorder and conduct disorder and associations between these conditions and prenatal stress,^14,48^ it may be that elevations in fetal steroid activity are not specific to autism. However, even if there is no specificity for elevations in autism, this would not necessarily detract from the significance of the results, as the elevations in autism may be more specific to the phenotype when observed in combination with other risk factors for autism that steroid hormones abnormalities may interact with in early development. Finally, given the evidence for positive correlations between all Δ4 steroids and non-Δ4 steroid hormones such as cortisol in early fetal development, future work should investigate interactions between hypothalamic-pituitary-adrenal and hypothalamic-pituitary-gonadal axes in early brain development.

In conclusion, we report the first direct evidence that steroidogenic activity is elevated in fetal development of those who later receive diagnoses on the autism spectrum. These results raise new questions for understanding a wide array of other observations about the early development of autism, through their interactions with early fetal steroidogenic abnormalities and provide initial support for the importance of fetal steroid hormones as important epigenetic fetal programming mechanisms for autism.

## Figures and Tables

**Table 1:** Sample characteristics

*Group variable*	*Controls (*n*=217)*	*Childhood autism (F84.0;* n*=41)*	*Asperger syndrome (F84.5;* n*=29)*	*PDD-NOS (F84.1, F84.8, F84.9;* n*=581)*	*ANOVA F(3,341)*	P*-value*
Maternal age (years)	33.59 (5.03)	34.12 (5.89)	33.52 (5.34)	32.32 (6.15)	1.11	0.34
Paternal age (years)	35.59 (6.53)	37.29 (7.21)	33.69 (6.06)	35.03 (8.13)	1.48	0.21
Birth weight (g)	3529.45 (662.01)	3659.75 (802.10)	3432.41 (622.77)	3446.63 (543.06)	0.93	0.42
Gestational age at sampling (weeks)	14.94 (1.95)	15.02 (1.33)	15.41 (1.74)	15.06 (1.71)	0.56	0.63
Apgar (% of sample w/ score >6)	99%	95%	100%	100%	—	—
Storage time (years)	14.78 (1.64)	14.63 (1.84)	14.41 (1.80)	14.84 (1.63)	0.54	0.65

Abbreviations: ANOVA, analysis of variance; PDD-NOS, pervasive developmental disorder not otherwise specified.

Means and s.d. (within parentheses) are given for each group along with the F-statistic and *P*-value from an ANOVA testing for between-group differences.

**Table 2:** Descriptive statistics for each hormone and each group

	*Control (*n*=217)*	*All autism (F84.0, F84.1, F84.5, F84.8, F84.9;* n*=128)*	*Childhood autism (F84.0;* n*=41)*	*Asperger syndrome (F84.5;* n*=29)*	*PDD-NOS (F84.1, F84.8, F84.9;* n*=58)*
Progesterone	49.2085 (26.7755)	59.2323 (33.3872)	65.0100 (33.8850)	60.6800 (37.0477)	54.4243 (30.9040)
17α-Hydroxy-progesterone	1.5056 (0.5494)	1.6853 (0.5988)	1.6722 (0.5246)	1.7059 (0.5331)	1.6843 (0.6826)
Androstenedione	0.9701 (0.4793)	1.1223 (0.7282)	1.0590 (0.4866)	1.0310 (0.3673)	1.2128 (0.9662)
Testosterone	0.7850 (0.3681)	0.8523 (0.4009)	0.8224 (0.4080)	0.9590 (0.4568)	0.8202 (0.3625)
Cortisol	0.3384 (0.1748)	0.3739 (0.2115)	0.3637 (0.1564)	0.3272 (0.1245)	0.4050 (0.2721)

Abbreviations: PDD-NOS, pervasive developmental disorder not otherwise specified.

Mean and s.d. values are in units of nmoL/L.
