# Supplementary figures and images for: Elevated fetal steroidogenic activity in autism
Source: Mol Psychiatry. 2014 Jun 3;20(3):369–76. doi: 10.1038/mp.2014.48 (PMC4184868; doi:10.1038/mp.2014.48)

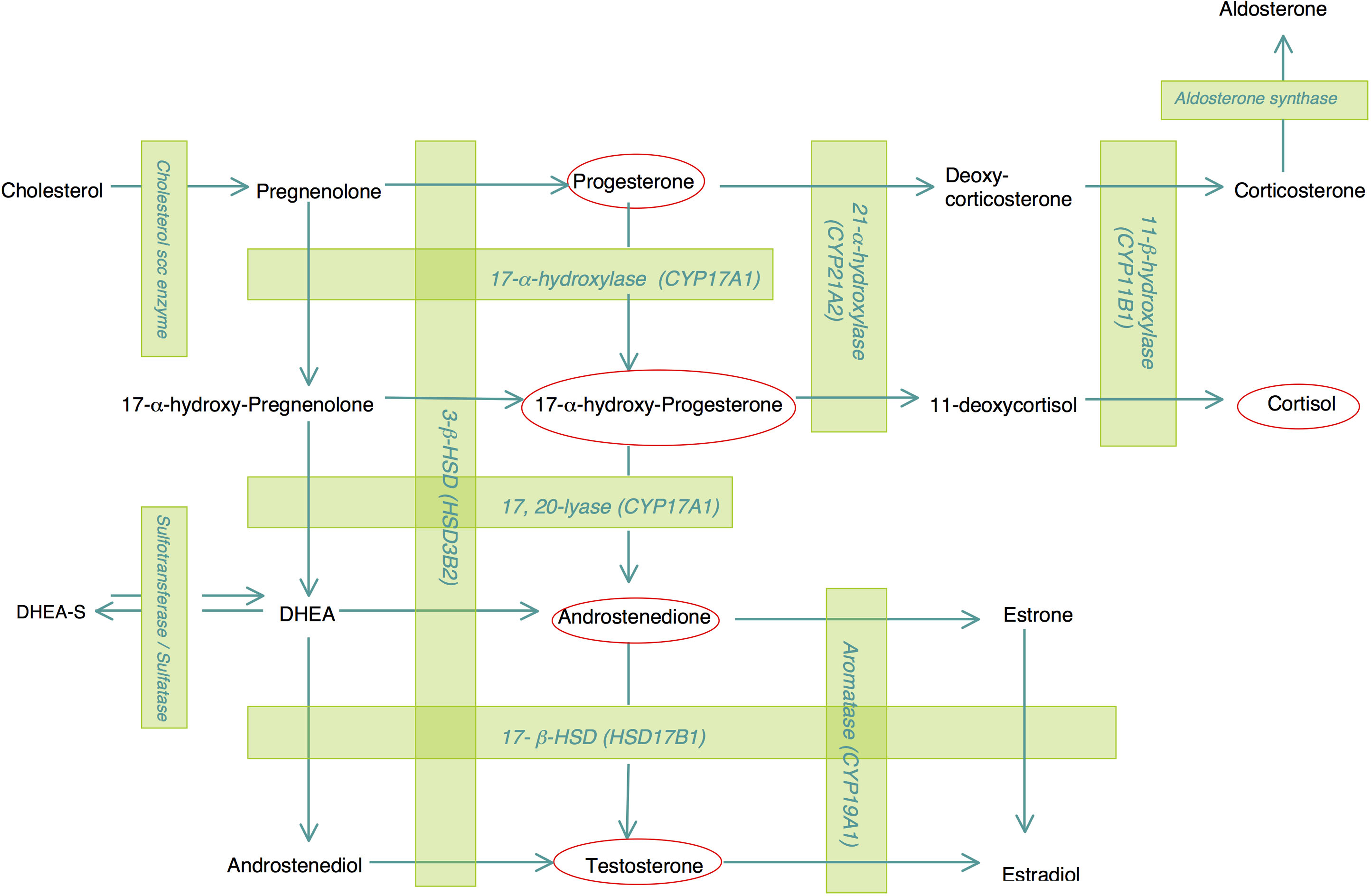

Supplement: Supplementary Figure 1 [file mp201448x1.tif]

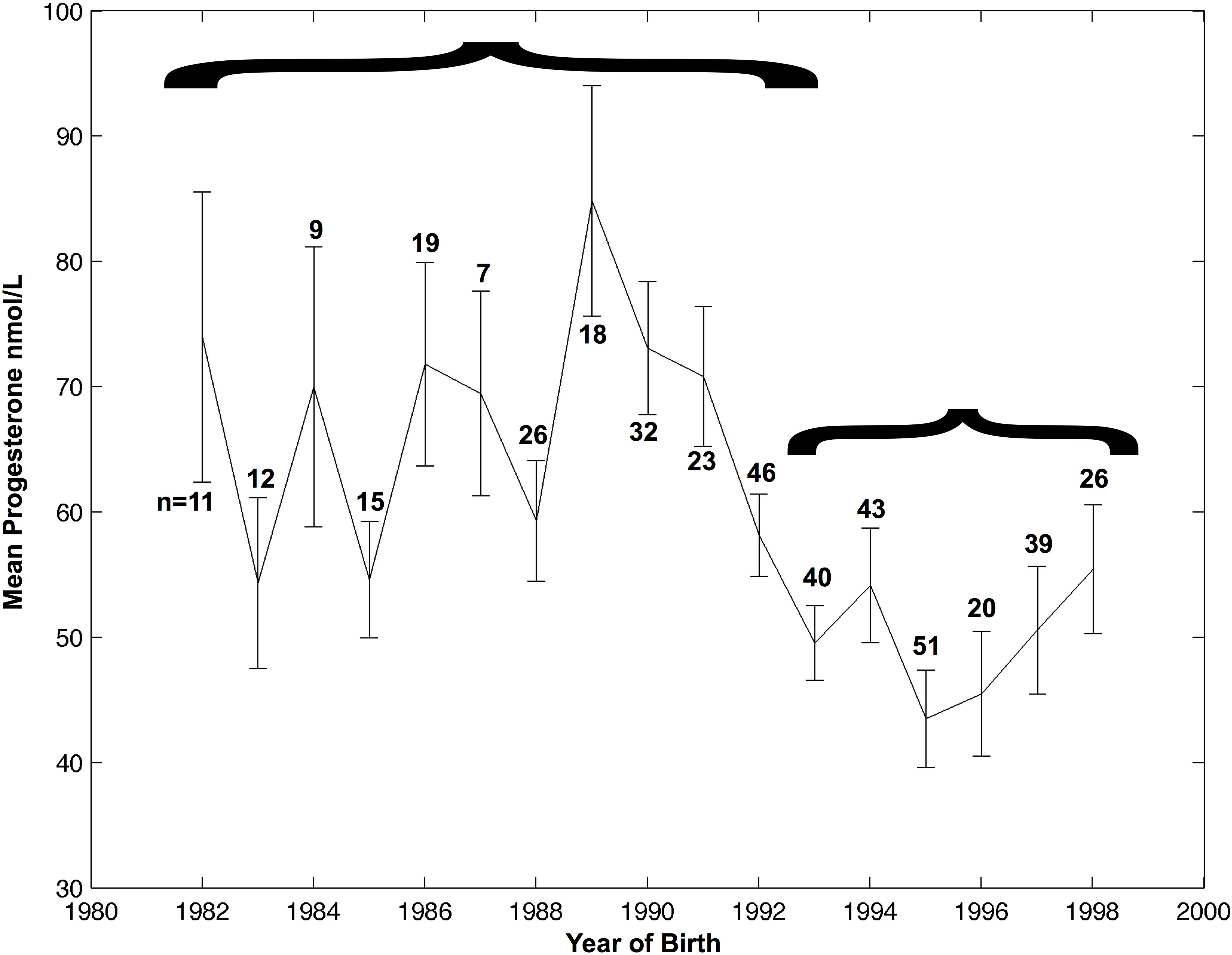

Supplement: Supplementary Figure 2 [file mp201448x2.tif]

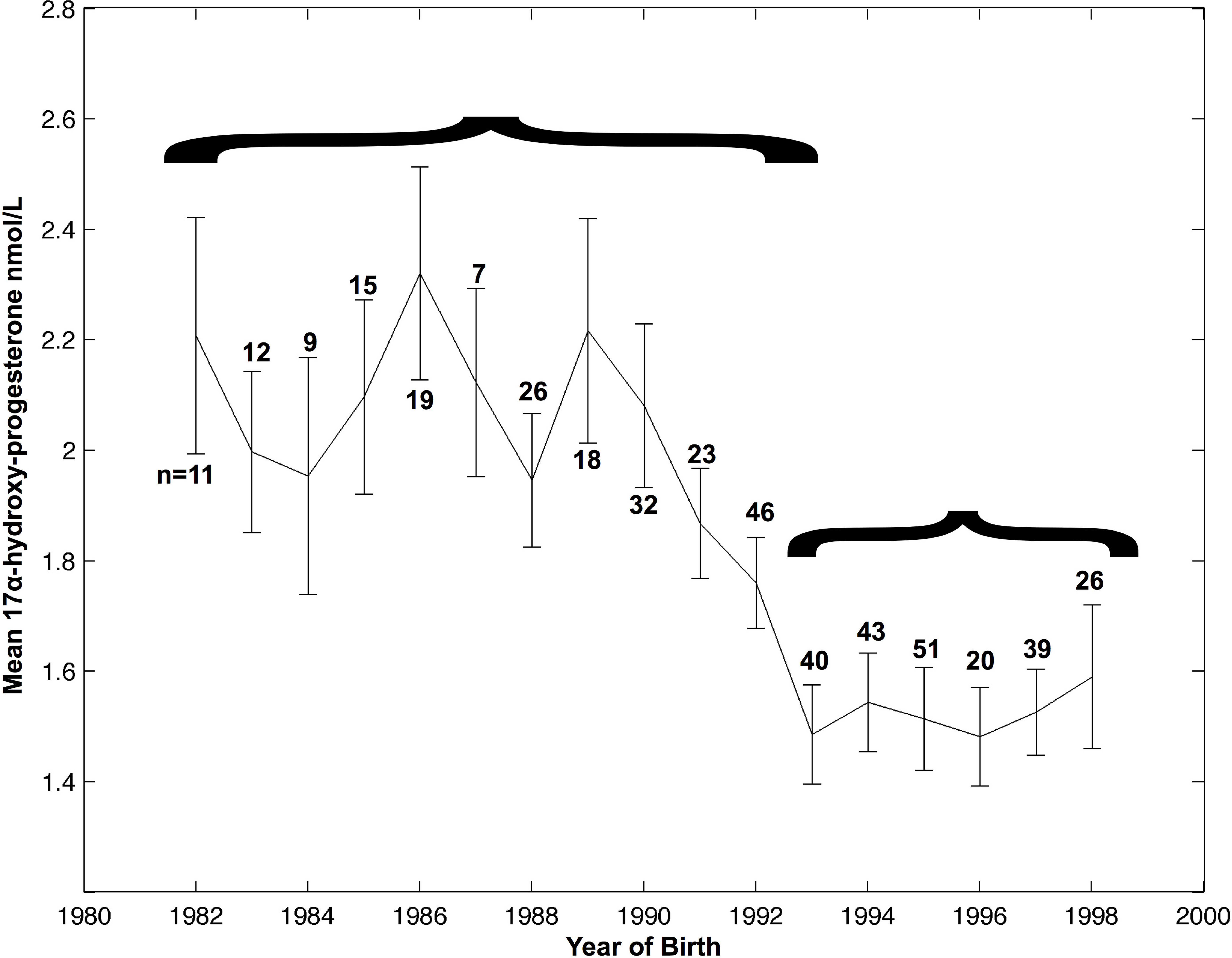

Supplement: Supplementary Figure 3 [file mp201448x3.tif]

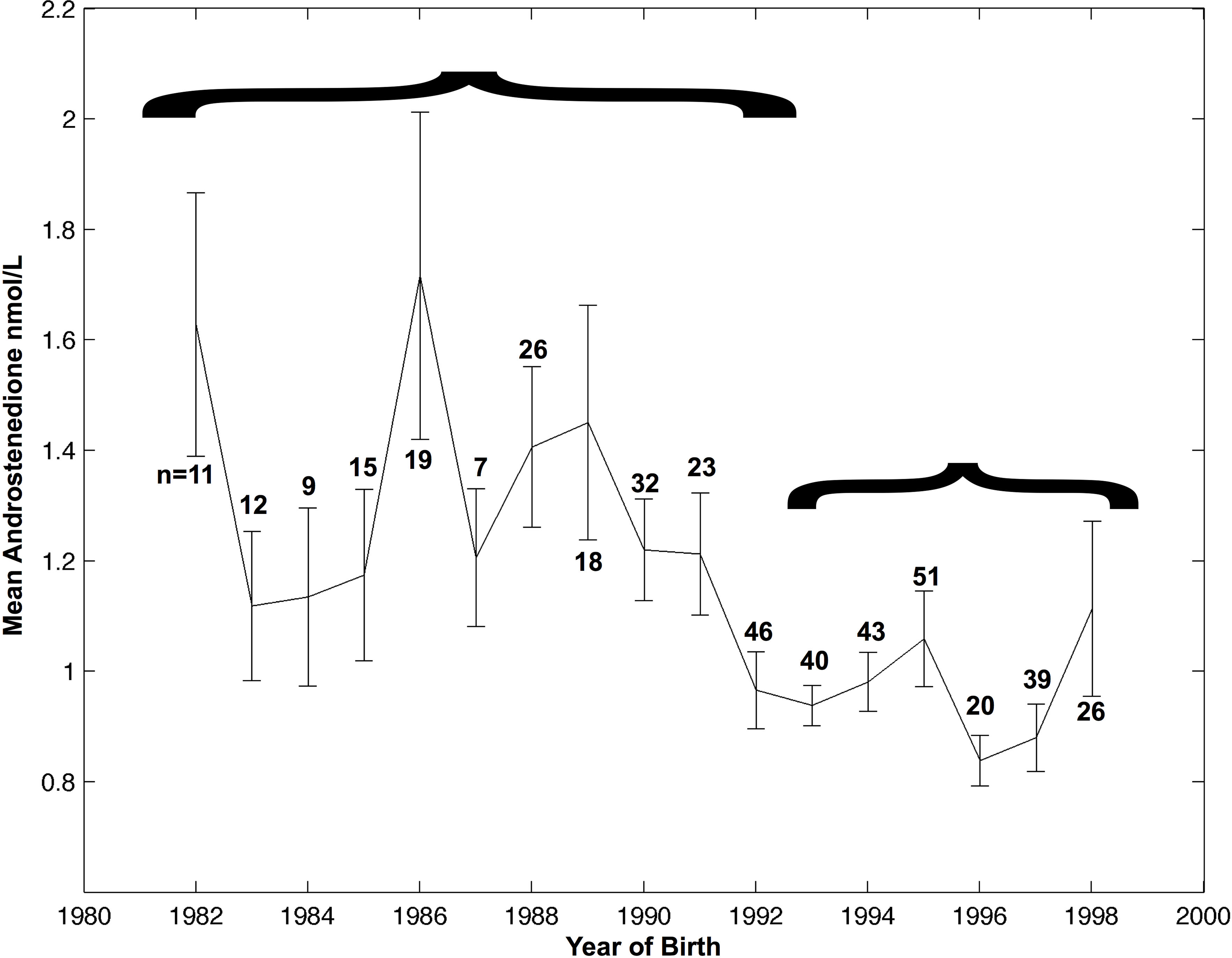

Supplement: Supplementary Figure 4 [file mp201448x4.tif]

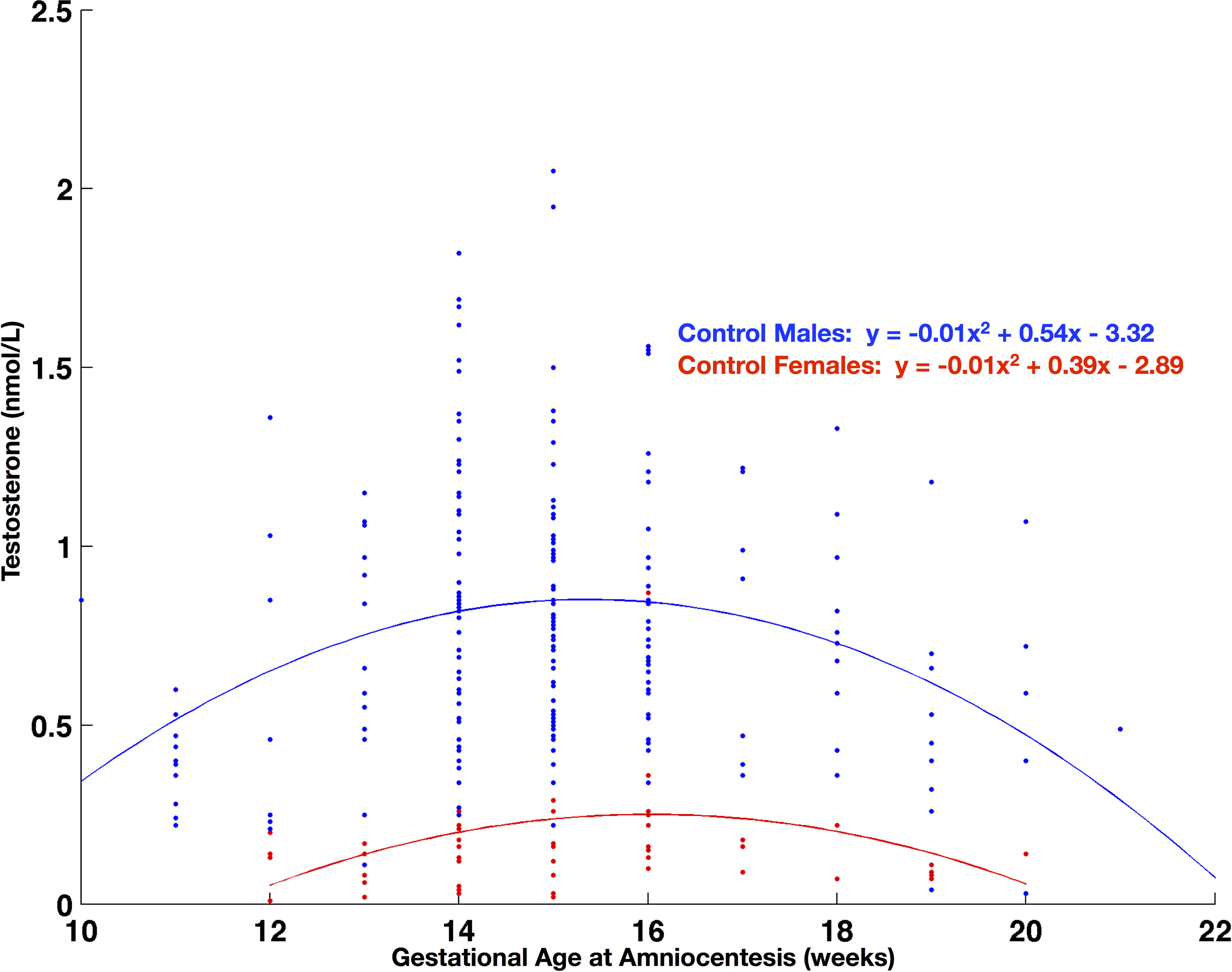

Supplement: Supplementary Figure 5 [file mp201448x5.tif]

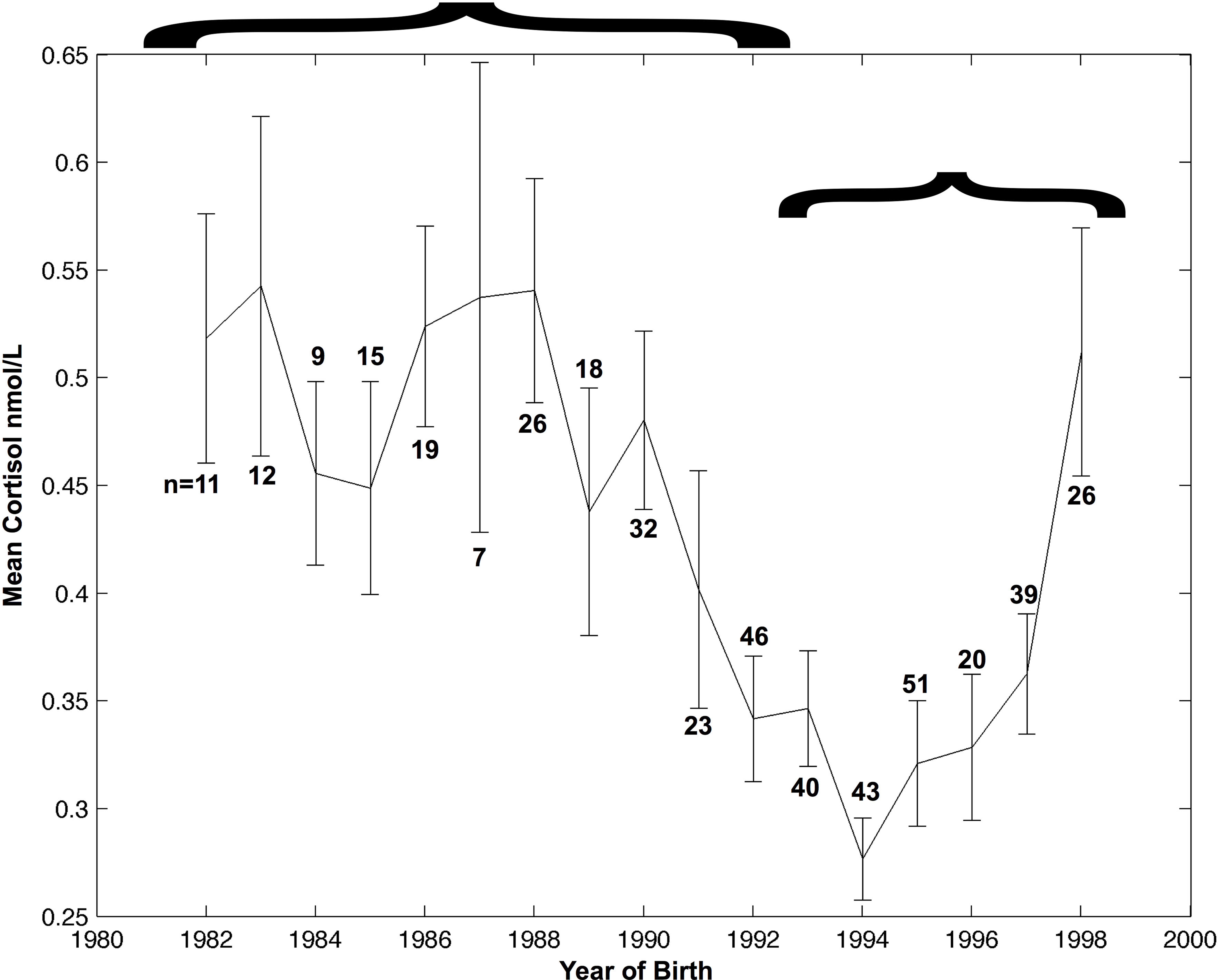

Supplement: Supplementary Figure 6 [file mp201448x6.tif]

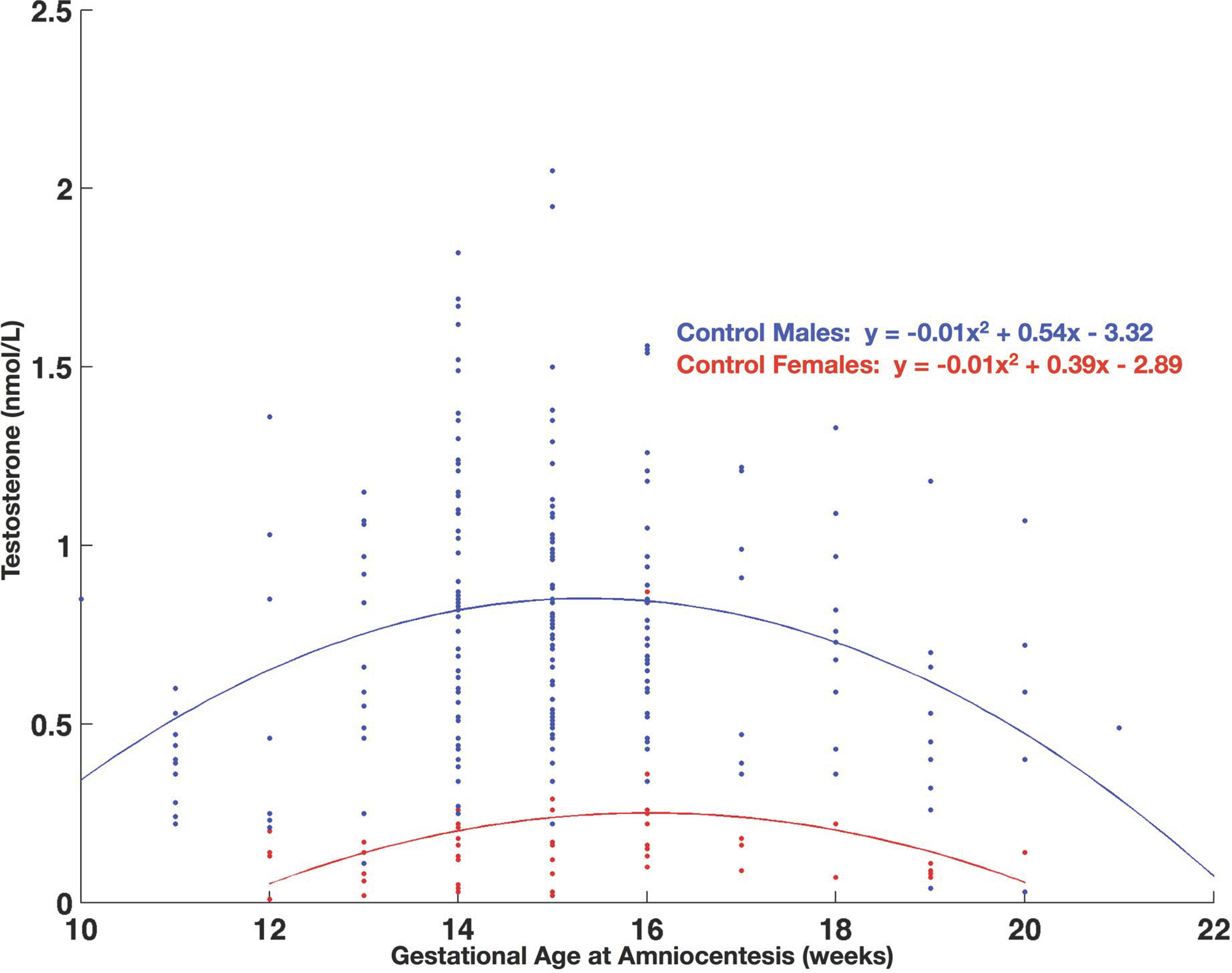

Supplement: Supplementary Figure 7 [file mp201448x7.tif]
