# Supplementary material for: Elevated fetal steroidogenic activity in autism
Source: Mol Psychiatry. 2014 Jun 3;20(3):369–76. doi: 10.1038/mp.2014.48 (PMC4184868; doi:10.1038/mp.2014.48)
Supplement: Supplementary Information [file mp201448x8.doc]

**Supplementary Material**

**Initial Quality Control Analyses of Samples from Typically-Developing Controls**

Changes in analyte concentration levels over prolonged storage times is a known issue[^1^](#_ENREF_1) and these analyses were conducted to examine whether such issues were apparent in the dataset. In addition, we were aware of a difference in the way the samples were stored pre- and post-1993. Caps used to seal the storage tubes were systematically changed in 1993 to one with a more effective cap for sealing the tube. Close visual inspection of pre-1993 samples showed evidence of evaporation (e.g., signs of change in the original stored volume). Thus, the concentration levels of various analytes in the pre-1993 tubes may have been changed in the remaining solvent[^1^](#_ENREF_1). There were no signs of evaporation in the post-1993 samples. Given these two issues, these quality check analyses were carried out on non-autistic samples to test for signs of storage-time-dependent changes (i.e., generalized upward or downward shifts in hormone concentration in pre- vs. post-1993 cohorts). Cases with a diagnosis of autism were not analyzed in these quality control analyses, because our main hypotheses predicted abnormalities in concentration levels, and it would be impossible to ascertain whether abnormalities were due to sample degradation issues or true abnormalities associated with having a diagnosis. In addition to comparing the 1982-1992 cohort to the 1993-1999 cohort, we further compared the results from each cohort to concentration levels from a meta-analysis of the literature on amniotic fluid testosterone levels as a reference check against both cohorts. The idea here was that values closer to the meta-analytic reference values were less likely to be influenced by storage-time-dependent changes.

As with analyses in the main paper, only those individuals where the assay was run twice and the difference between duplicate assays was less than 3 standard deviations were used in this analysis. We also excluded individuals who had values of 0 (i.e., where hormone concentration was below the level of sensitivity detectable to the assay) and excluded extreme outlying values greater than the 99^th^ percentile of the entire sample (1982-1999).

We found that all 5 hormones showed generalized increases in concentrations in the 1982-1992 cohort compared to 1993-1999 cohort (all p<0.0016) (see Supplementary Table 1). These increases are shown in Supplementary Figures 2-6 and resemble a step-function. These abnormalities are consistent with observations of evaporation in the tubes pre-1993 that coincide with the systematic change in the way the caps sealed tubes for storage.

To further examine storage-time-dependent changes in the 1982-1992 cohort, we compared testosterone concentrations from both the 1982-1992 and 1993-1999 cohorts to a reference value obtained via a meta-analysis of studies reporting testosterone concentrations from amniotic fluid sampled during amniocentesis. For the meta-analysis, we conducted a systematic literature search using PubMed, ScienceDirect, Web of Knowledge and Scopus databases for the keywords ‘fetal testosterone’, ‘amniotic fluid testosterone’, ‘prenatal sex hormone levels’, ‘fetal sex’, and ‘reference levels’. Inclusion criteria were that the amniotic fluid testosterone was measured by standard radioimmunoassays (RIA) or liquid chromatography mass spectrometry. and that the paper reported the mean or median statistic as well as the number of samples analyzed (n). Where different units of measure were reported (i.e. ng/L, ng/dL, pmol/L), we converted them into the unit of measure we used (nmol/L). We also did not include any of the studies published by our group and instead replaced them with one summary statistic computed on our entire database of all individuals with an amniotic fluid testosterone sample. This was done to eliminate redundancy within our papers, because there is some degree of overlap in the participants reported across our various papers[^2-8^](#_ENREF_2). A total of 18 studies were found. Seven of these studies had to be excluded for the following reasons: 2 studies did not report any measures of central tendency and only reported ranges; 2 studies used modified RIA protocols and its comparability with more standard RIAs could not be substantiated; 1 study used time-resolved fluorescence immunoassay; 1 study did not specify the assay method; 1 study reported only free-testosterone levels. Including our Cambridge summary statistic with these studies, we were left with a total of 12 studies for the meta-analysis. In the final analysis we computed a reference value for testosterone by calculating a weighted measure of central tendency (either mean or median).

Supplementary Table 2 reports the studies included in the meta-analysis[^2-19^](#_ENREF_2). The meta-analysis weighted mean for testosterone amongst the 9 studies reporting mean values for male samples was 0.8244 nmol/L. Testosterone concentration from the 1982-1992 cohort (mean = 0.9036 nmol/L, SD = 0.4071) was significantly different from this meta-analysis weighted mean (t(217)=2.875, p=0.004). Similar inferences were obtained, using bootstrapping (100,000 resamples) to compute the mean and derive the 95% bias-corrected and accelerated confidence intervals. We found that the meta-analysis reference mean was less than the lower bound confidence interval (95% BCa CI = 0.8515 to 0.9585). In contrast, there was no difference in mean level of testosterone in the 1993-1999 cohort (mean = 0.7865 nmol/L, SD = 0.3670) compared to the meta-analysis weighted mean (t(218)=-1.529, p=0.128). Similarly, using bootstrapping (100,000 resamples), we found that the meta-analysis reference mean was within the 95% BCa confidence intervals (95% BCa CI = 0.7396 to 0.8368).

From the 3 studies reporting the median value, the meta-analysis weighted median was 0.7772 nmol/L. Bootstrapping was again used to compared this meta-analysis reference median to the medians from both the 1982-1992 and 1993-1999 cohorts. We found that the reference median was below the lower bound 95% BCa confidence interval for the 1982-1992 cohort (95% BCa CI = 0.79 to 0.8950) but was within the confidence intervals for the 1993-1999 cohort (95% BCa CI = 0.69 to 0.83).

From these analyses we can see that only the 1993-1999 cohort had testosterone concentrations that were similar to the reference mean and median values from the meta-analysis, whereas the concentration levels for the 1982-1992 cohort were consistently above the reference mean and median values. As a result of these inferences, all main analyses reported in the main manuscript only included data of individuals from the 1993-1999 cohort.

Finally, there were other independent reasons for why using the 1993-1999 cohort minimizes other sources of noise variation. First, after 1993 the timing of amniocentesis in Denmark was standardized using ultrasound as a way of marking gestational age, whereas such a method was not consistently used before 1993. Second, diagnostic information after 1993 (particularly for autism) became much more reliable. ICD-8 was used in Denmark until the end of 1993 and then switched to ICD-10[^20^](#_ENREF_20). In the ICD classification, the definition of infantile autism changed from psychosis proto-infantalis in ICD-8 (code 299.00) to childhood autism in ICD-10 (code F84.0). The other autism diagnoses, however, were listed in more specific diagnostic entities in ICD-10. The transition from ICD-8 to ICD-10 is considered to have improved considerably the definition of the diagnostic entities of autism[^21^](#_ENREF_21).

**Supplementary Figure 1:** **Steroidogenic Biosynthesis Pathways**

*Circled in red are the hormones analyzed in the amniotic fluid from the current study. In italics are the enzymes that catalyze conversion of hormones and in parentheses are the gene name for producing each enzyme.*

**Supplementary Figure 2:** **Progesterone concentrations in typically-developing Controls across each year of birth**

*This graph plots the mean progesterone concentration (+/- 1 standard error of the mean) for each year of birth from 1982 to 1998. The number next to each plot is the sample size for that year. The curly brackets are used to denote the 1982-1992 and 1993-1999 cohorts.*

**Supplementary Figure 3:** **17α-hydroxy-progesterone concentrations in typically-developing Controls across each year of birth**

*This graph plots the mean 17α-hydroxy-progesterone concentration (+/- 1 standard error of the mean) for each year of birth from 1982 to 1998. The number next to each plot is the sample size for that year. The curly brackets are used to denote the pre-1981-1992 and 1993-1999 cohorts.*

**Supplementary Figure 4:** **Androstenedione concentrations in typically -developing Controls across each year of birth**.

*This graph plots the mean androstenedione concentration (+/- 1 standard error of the mean) for each year of birth from 1982 to 1998. The number next to each plot is the sample size for that year. The curly brackets are used to denote the pre-1981-1992 and 1993-1999 cohorts.*

**Supplementary Figure 5:** **Testosterone concentrations in typically-developing Controls across each year of birth**

*This graph plots the mean testosterone concentration (+/- 1 standard error of the mean) for each year of birth from 1982 to 1998. The number next to each plot is the sample size for that year. The curly brackets are used to denote the pre-1981-1992 and 1993-1999 cohorts.*

**Supplementary Figure 6:** **Cortisol concentrations in typically-developing Controls across each year of birth.**

*This graph plots the mean cortisol concentration (+/- 1 standard error of the mean) for each year of birth from 1982 to 1998. The number next to each plot is the sample size for that year. The curly brackets are used to denote the pre-1981-1992 and 1993-1999 cohorts.*

**Supplementary Figure 7: Testosterone levels as a function of gestational age at amniocentesis and illustration of the sex difference in testosterone amongst typically-developing Controls from the 1993-1999 cohort.**

This plot shows testosterone concentration levels as a function of gestational age at amniocentesis. The data for males (blue) generally corresponds to a second-order polynomial fit (inverted U-shape), and peaks around 14-16 weeks gestation. This corresponds well to what is known about the early prenatal surge in humans from work looking at fetal serum levels (see [^22^](#_ENREF_22) and [^23^](#_ENREF_23) for more). This plot also clearly shows the substantial sex difference in testosterone levels between males (blue) and females (red) and the effect size is Cohen’s d = 1.71.

**Supplementary Table 1: Hormone concentrations of typically-developing Controls in the 1982-1992 vs. 1993-1999 year of birth cohorts**

*Mean and standard deviation values are in units of nmol/L.*

|  | **1982-1992**  **Mean (SD)** | **1993-1999**  **Mean (SD)** | ***t*(435) (p-value)** |
| --- | --- | --- | --- |
| **Progesterone**  1982-1992 n = 218  1993-1999 n = 219 | 66.3927 (29.1289) | 49.5539 (26.9780) | 6.2698  (8.72 x 10^-10^) |
| **17α-hydroxy-progesterone**  1982-1992 n = 218  1993-1999 n = 219 | 2.0043 (0.6836) | 1.5217 (0.5758) | 7.9818  (1.3 x 10^-14^) |
| **Androstenedione**  1982-1992 n = 218  1993-1999 n = 219 | 1.2577 (0.7102) | 0.9759 (0.4813) | 4.8562  (1.67 x 10^-6^) |
| **Testosterone**  1982-1992 n = 218  1993-1999 n = 219 | 0.9036 (0.4071) | 0.7864 (0.3670) | 3.1606  (0.0016) |
| **Cortisol**  1982-1992 n = 218  1993-1999 n = 219 | 0.4541 (0.2347) | 0.3477 (0.1991) | 5.1126  (4.76 x 10^-7^) |

**Supplementary Table 2: Studies included in the meta-analysis for testosterone.**

*** indicates that the central tendency measured by the median. Otherwise, central tendency is measured by the mean**

|  | **Males** | | **Females** | |
| --- | --- | --- | --- | --- |
| **Reference** | **N** | **Testosterone Concentration (nmol/L)** | **N** | **Testosterone Concentration (nmol/L)** |
| Bay et al., 2008 | 46 | 1.04 | 48 | 0.34 |
| Bergman et al., 2010 | 53 | 0.8 | 54 | 0.24 |
| Frasier et al., 1974 | 28 | 0.99 | 20 | 0.78 |
| Giles et al., 1974 | 38 | 0.61 | 19 | 0.21 |
| Judd et al., 1976 | 58 | 0.77 | 77 | 0.14 |
| Perera et al., 1987* | 185 | 0.71* | 166 | 0.13* |
| Robertson et al., 1980 | 62 | 0.55 | 35 | 0.21 |
| Rodeck et al., 1985 | 49 | 1.18 | 21 | 0.73 |
| Sarkar et al., 2007* | 133 | 0.85* | 131 | 0.28* |
| Wudy et al., 1999* | 45 | 0.83* | 32 | 0* |
| Zondek et al., 1977 | 31 | 0.58 | 23 | 0.16 |
| Cambridge FT database [^2-8^](#_ENREF_2) | 313 | 0.84 | 287 | 0.33 |

**References**

1. Kugler K, Hackl W, Mueller L, Fiegl H, Graber A, Pfeiffer R. The impact of sample storage time on estimates of association in biomarker discovery studies. *Journal of Clinical Bioinformatics* 2011; **1**(1)**:** 1-8.

2. Auyeung B, Baron-Cohen S, Ashwin E, Knickmeyer R, Taylor K, Hackett G. Fetal testosterone and autistic traits. *Br J Psychol* 2009; **100**(Pt 1)**:** 1-22.

3. Auyeung B, Baron-Cohen S, Chapman E, Knickmeyer R, Taylor K, Hackett G. Foetal testosterone and the child systemizing quotient. *European Journal of Endrocrinology* 2006; **155:** 123-130.

4. Auyeung B, Taylor K, Hackett G, Baron-Cohen S. Foetal testosterone and autistic traits in 18 to 24-month-old children. *Mol Autism* 2010; **1**(1)**:** 11.

5. Chapman E, Baron-Cohen S, Auyeung B, Knickmeyer R, Taylor K, Hackett G. Fetal testosterone and empathy: evidence from the empathy quotient (EQ) and the "reading the mind in the eyes" test. *Soc Neurosci* 2006; **1**(2)**:** 135-148.

6. Knickmeyer R, Baron-Cohen S, Raggatt P, Taylor K. Foetal testosterone, social relationships, and restricted interests in children. *J Child Psychol Psychiatry* 2005; **46**(2)**:** 198-210.

7. Knickmeyer RC, Wheelwright S, Taylor K, Raggatt P, Hackett G, Baron-Cohen S. Gender-typed play and amniotic testosterone. *Dev Psychol* 2005; **41**(3)**:** 517-528.

8. Lutchmaya S, Baron-Cohen S, Raggatt P. Foetal testosterone and eye contact in 12-month-old human infants. *Infant Behavior and Development* 2002; **25:** 327-335.

9. Bay K, Cohen AS, Jorgensen FS, Jorgensen C, Lind AM, Skakkebaek NE *et al.* Insulin-like factor 3 levels in second-trimester amniotic fluid. *The Journal of clinical endocrinology and metabolism* 2008; **93**(10)**:** 4048-4051.

10. Bergman K, Glover V, Sarkar P, Abbott DH, O'Connor TG. In utero cortisol and testosterone exposure and fear reactivity in infancy. *Horm Behav* 2010; **57**(3)**:** 306-312.

11. Frasier SD, Weiss BA, Horton R. Amniotic fluid testosterone: Implications for the prenatal diagnosis of congenital adrenal hyperplasia. *J Pediatr* 1974; **84:** 738-741.

12. Giles HR, Lox CD, Heine MW, Christian CD. Intrauterine fetal sex determonation by radioimmunoassay of amniotic fluid testosterone. *Gynecol Invest* 1974; **5**(5-6)**:** 317-323.

13. Zondek T, Mansfield MD, Zondek LH. Amniotic fluid testosterone and fetal sex determination in the first half of pregnancy. *British Journal of Obstetrics and Gynaecology* 1977; **84:** 714-716.

14. Judd HL, Robinson JD, Young PE, Jones OW. Amniotic fluid testosterone levels in midpregnancy. *Obstetrics and gynecology* 1976; **48**(6)**:** 690-692.

15. Robertson RD, Henniker AJ, Luttrell BM, Saunders DM. The prenatal determination of fetal sex: amniotic fluid testosterone as a preliminary screening test. *Eur J Obstet Gynecol Reprod Biol* 1980; **10**(2)**:** 77-81.

16. Rodeck CH, Gill D, Rosenberg DA, Collins WP. Testosterone levels in midtrimester maternal and fetal plasma and amniotic fluid. *Prenatal diagnosis* 1985; **5**(3)**:** 175-181.

17. Sarkar P, Bergman K, Fisk NM, O'Connor TG, Glover V. Amniotic fluid testosterone: relationship with cortisol and gestational age. *Clin Endocrinol (Oxf)* 2007; **67**(5)**:** 743-747.

18. Perera DM, McGarrigle HH, Lawrence DM, Lucas M. Amniotic fluid testosterone and testosterone glucuronide levels in the determination of foetal sex. *J Steroid Biochem* 1987; **26**(2)**:** 273-277.

19. Wudy SA, Dorr HG, Solleder C, Djalali M, Homoki J. Profiling steroid hormones in amniotic fluid of midpregnancy by routine stable isotope dilution/gas chromatography-mass spectrometry: reference values and concentrations in fetuses at risk for 21-hydroxylase deficiency. *The Journal of clinical endocrinology and metabolism* 1999; **84**(8)**:** 2724-2728.

20. Lauritsen M, Jørgensen M, Madsen K, Lemcke S, Toft S, Grove J *et al.* Validity of Childhood Autism in the Danish Psychiatric Central Register: Findings from a Cohort Sample Born 1990–1999. *Journal of autism and developmental disorders* 2010; **40**(2)**:** 139-148.

21. Lauritsen M, Pedersen C, Mortensen P. The incidence and prevalence of pervasive developmental disorders: a Danish population-based study. *Psychological medicine* 2004; **34**(07)**:** 1339-1346.

22. Hines M. Sex steroids and human behavior: prenatal androgen exposure and sex-typical play behavior in children. *Annals of the New York Academy of Sciences* 2003; **1007:** 272-282.

23. Smail PJ, Reyes FI, Winter JSD, Fairman C. The fetal hormonal environment and its effect on the morphogenesis of the genital system. In: Kogan SJ, Hafez ESE (eds). *Pediatric andrology*. Martinus Nijhoff: The Hague, 1981, pp 9-19.
